# Supplementary material for: AI, ageing and brain-work productivity: Technological change in professional Japanese chess
Source: PLoS One. 2024 May 2;19(5):e0299889. doi: 10.1371/journal.pone.0299889 (PMC11065245; doi:10.1371/journal.pone.0299889)
Supplement: S1 File — (PDF) [file pone.0299889.s001.pdf]

S1 Supporting Information.

S1 Fig 1. Change in player's productivity according to ages.

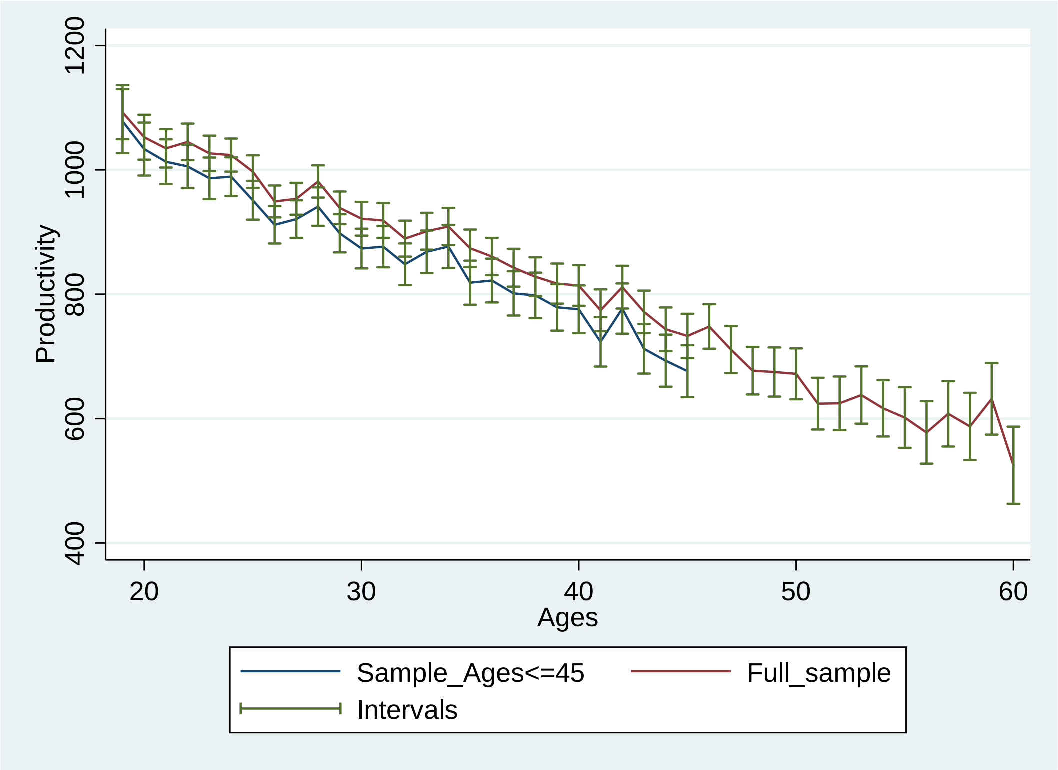

Note: The productivity index is calculated in the way of Bertoni et al. (2015, p.48).

S1 Fig 2 (1). Marginal effects used to illustrate Figure 7.

Effect of ages on player's productivity according to different debut ages. 1968-1989

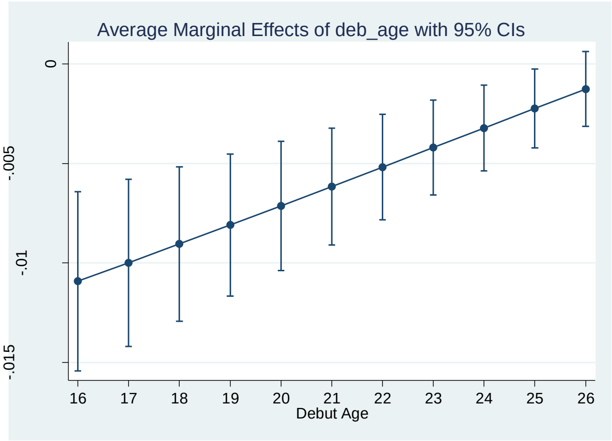

S1 Fig 2 (2). Marginal effects used to illustrate Figure 7.

Effect of ages on player's productivity according to different debut ages. 1990-2012

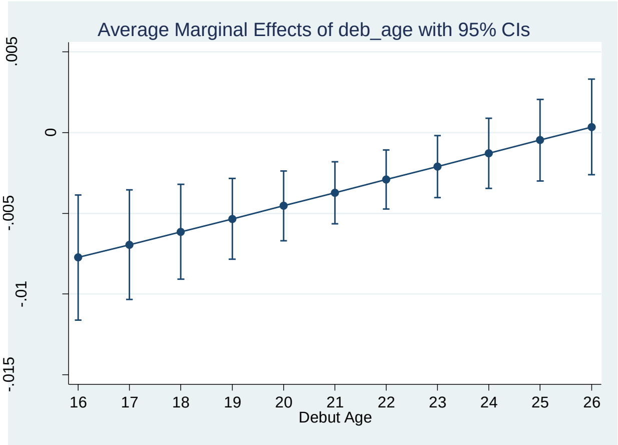

S1 Fig 2 (3). Marginal effects used to illustrate Figure 7.

Effect of ages on player's productivity according to different debut ages. 2013-2019

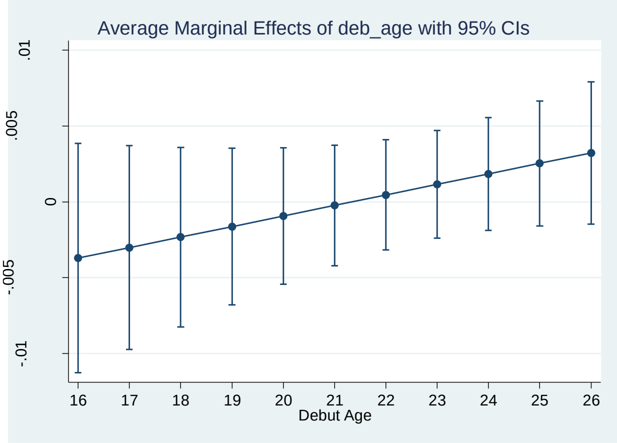

S1 Fig 3 (1). Marginal effects used to illustrate Figure 8.

Effect of ages on player's productivity according to different debut ages. 1968-1989

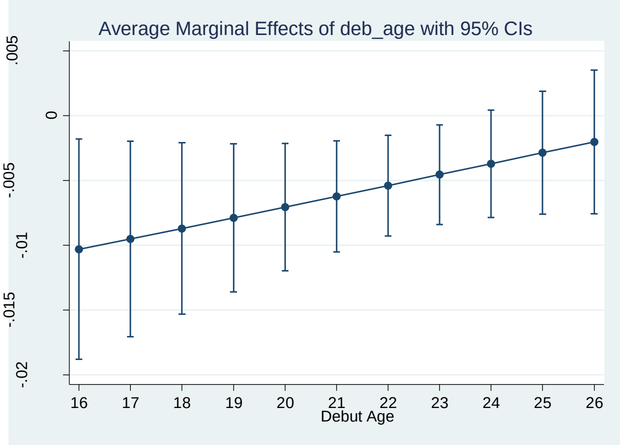

S1 Fig 3 (2). Marginal effects used to illustrate Figure 8.

Effect of ages on player's productivity according to different debut ages. 1990-2012

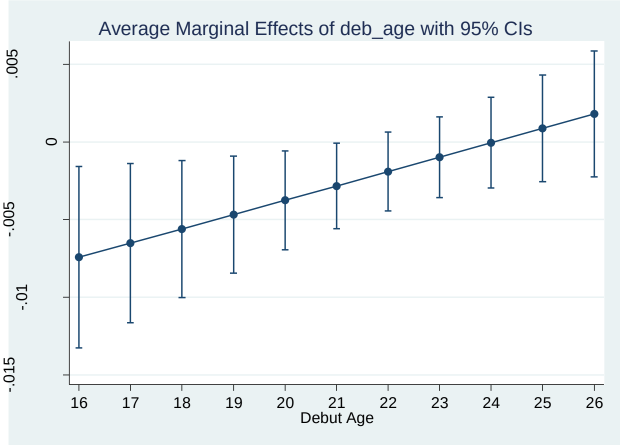

S1 Fig 3 (3). Marginal effects used to illustrate Figure 8.

Effect of ages on player's productivity according to different debut ages. 2013-2019

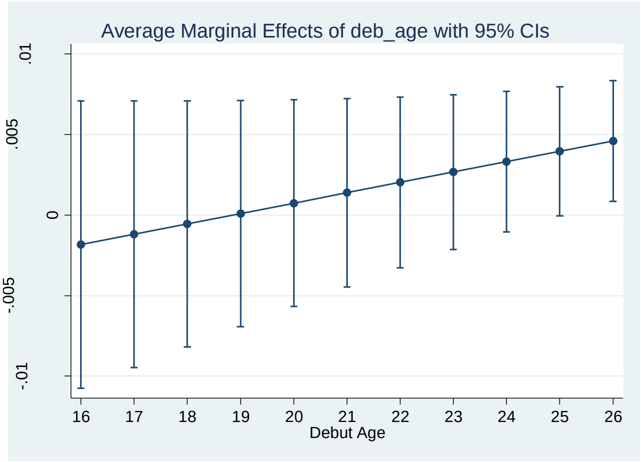

S1 Fig 4 (1). Marginal effects used to illustrate Figure 9.

Effect of ages on player's productivity according to different ages. 1968-1989

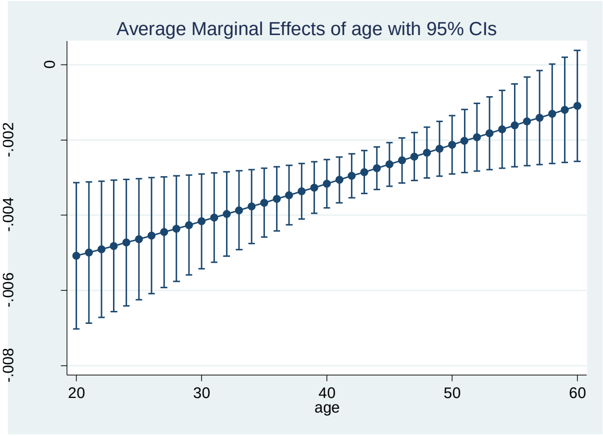

S1 Fig 4 (2). Marginal effects used to illustrate Figure 9.

Effect of ages on player's productivity according to different ages. 1990-2012

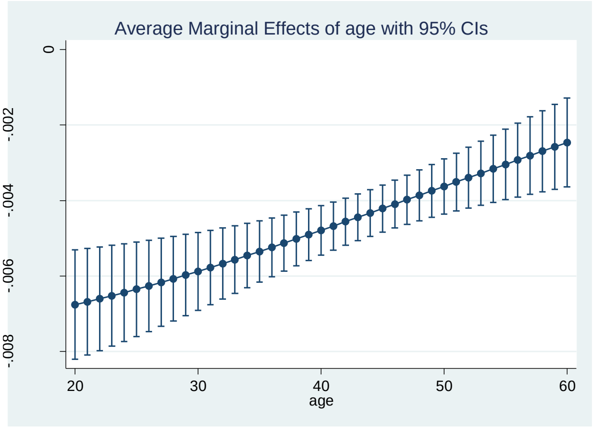

S1 Fig 4 (3). Marginal effects used to illustrate Figure 9.

Effect of ages on player's productivity according to different ages. 2013-2019

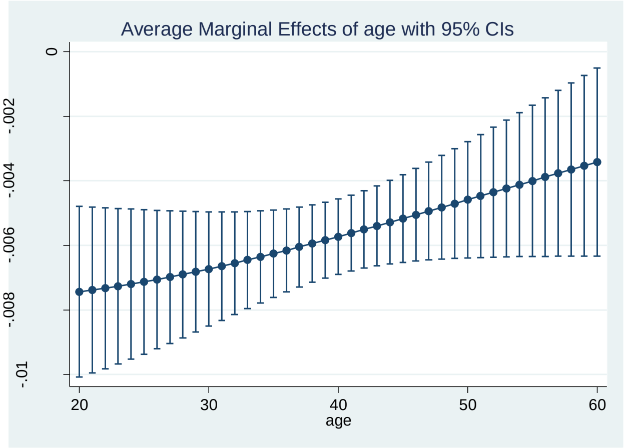

S1 Fig 5 (1). Marginal effects used to illustrate Figure 10.

Effect of ages on player’s productivity according to different ages. 1968-1989

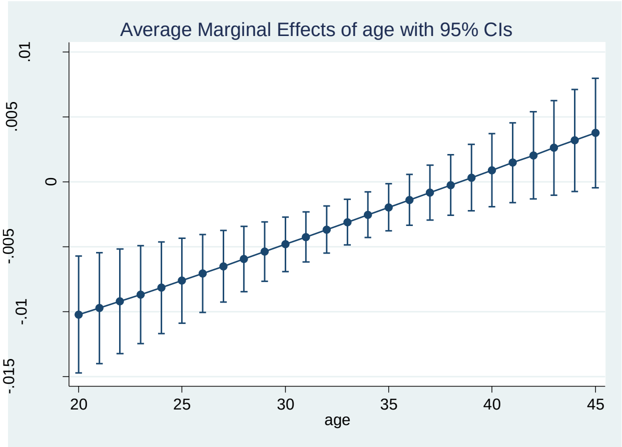

S1 Fig 5 (2). Marginal effects used to illustrate Figure 10.

Effect of ages on player’s productivity according to different ages. 1990-2012

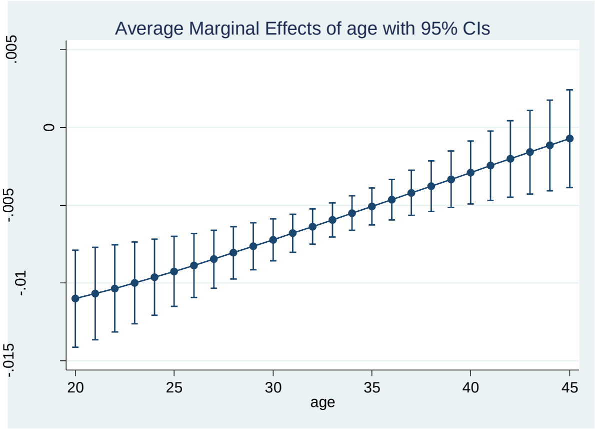

S1 Fig 5(3). Marginal effects used to illustrate Figure 10.

Effect of ages on player’s productivity according to different ages. 2013-2019

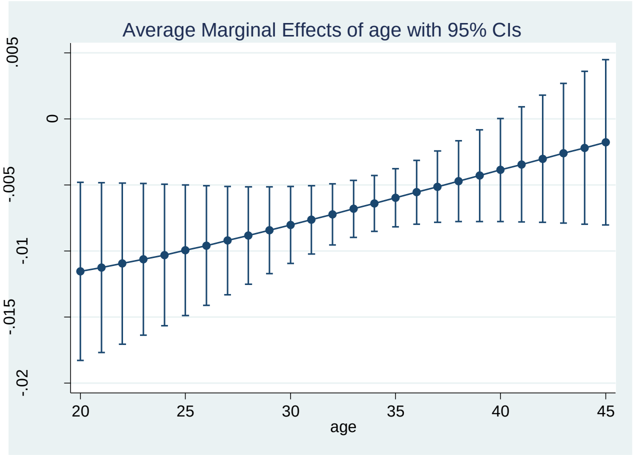

S1 Fig 6(1). Marginal effects used to illustrate Figure 11.

Effect of ages on player's productivity according to different ages. 1968-1989

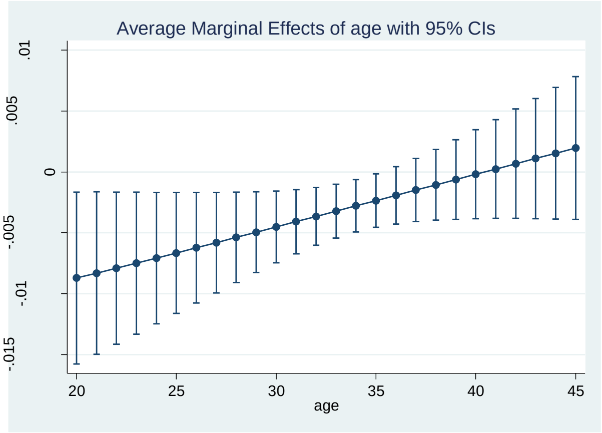

S1 Fig 6 (2). Marginal effects used to illustrate Figure 11.

Effect of ages on player's productivity according to different ages. 1990-2012

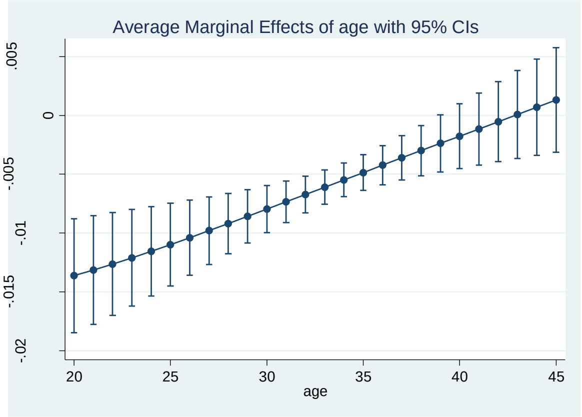

S1 Fig 6 (3). Marginal effects used to illustrate Figure 11.

Effect of ages on player's productivity according to different ages. 2013-2019

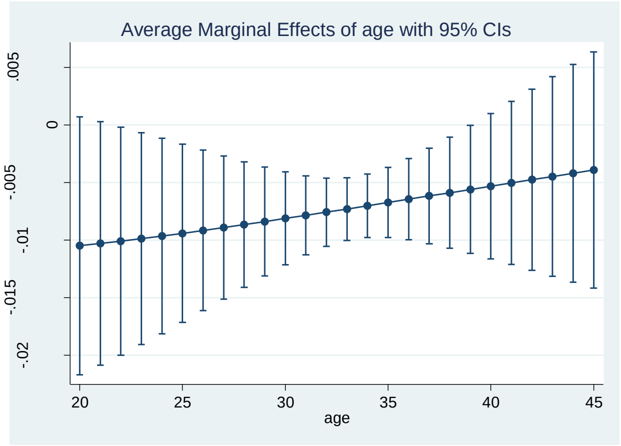

S1 Fig 7 (1). Marginal effects used to illustrate Figure 12.

Effect of ages on player's productivity according to different ages. 1968-1989

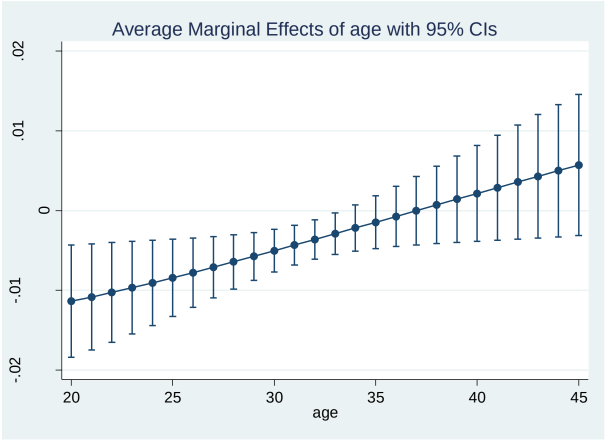

S1 Fig 7 (2). Marginal effects used to illustrate Figure 12.

Effect of ages on player's productivity according to different ages. 1990-2012

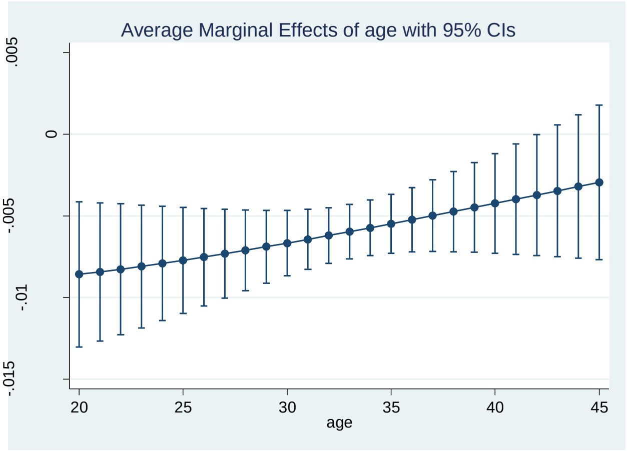

S1 Fig 7(3). Marginal effects used to illustrate Figure 12.

Effect of ages on player's productivity according to different ages. 2013-2019
