# Supplementary material for: AI, ageing and brain-work productivity: Technological change in professional Japanese chess
Source: PLoS One. 2024 May 2;19(5):e0299889. doi: 10.1371/journal.pone.0299889 (PMC11065245; doi:10.1371/journal.pone.0299889)
Supplement: Attachment — Submitted filename: PONE-D-23-24861_review.pdf [file pone.0299889.s002.pdf]

In this review I will always use the word “Chess” to refer to “international chess”, and the word “Shogi” to refer to “Japanese professional chess”.

This is an enjoyable paper, especially for a Chess aficionado like me, and for anyone interested in the impact of AI (or algorithms based on Machine Learning) in the present and future of society. Outside of board games/sports, the main taking from the paper is that the current state of AI is capable of reducing the dispersion of labor productivity, specifically in activities of intellectual nature. With the aid of computers, the performance of high-skilled workers will be similar, even when one worker is more productive than the other without that aid.

However, the paper needs some clarifications and precision of concepts and terms, a few changes in the methodological section, and some polish in the exposition.

Clarifications:

- Through all the paper, the phrase “the novel setting” is used. The authors must clarify what do they mean by that, or find a different wording.
- Page 2 line 26: “diffusion of artificial intelligence (AI) reduces innate ability”. I think that the authors really meant to say “diffusion of artificial intelligence (AI) reduces the impact of innate ability in player’s performance”.
- Authors should be more explicit on which of their finding are specific to board games as sports (Chess,Shogi,Go,etc.), to Shogi in particular, or to intellectual labor in general. For example, finding (1) in the Abstract could be extrapolated to the labor market, but I am not so sure about findings (2)-(4). After all, these are deterministic and finite games, the outcomes of which lack any ethical implication.
- Philosophizing a bit, I would say that AI might be more beneficial for older workers (not less) in many areas. Taking Chess as an example, we can argue that as players get older they tend to assess positions better due to experience, but they get slower calculating variations. That is, as “judgment” and “experience” increases, the “raw brain power” decreases. However, in board sports the use of AI *during* the game is *cheating*, but for many intellectual tasks it is *desirable* and even *encouraged*. While current AI cannot substitute human “judgment” and “experience” in many areas, it can greatly help with the “raw brain power”. Hence I argue that, in many examples, AI may be a more efficient tool in the hands of an experienced worker.
- Page 3 line 51: “However, the level of AI is almost equivalent”. I guess it was meant to say “However, the level of AI <u>was</u> almost equivalent” (at the time of Watanabe’s prediction).
- Page 4 line 54: “games against AI”. Shouldn’t it say “games against other human players?”.
- Page 4 lines 68-69: “The recent development of AI technology allows it to replicate the human brain and, therefore, would replace brain work.” I strongly disagree. Current AI gives us only the *illusion* of intelligence. It excels at pattern recognition, but it cannot *reason*, and does not understand *concepts*. For example, given thousands images to train, AI can distinguish a horse form a unicorn. In contrast, a

child can figure that "a unicorn is simply a horse with a horn" with less than 10 images. Current AI cannot figure out that simple fact. In Chess/Shogi AI *finds* good strategies, but so far only humans can rationalize *why* those strategies work, and apply them accordingly. Therefore, in the near future, AI will replace only "*some intellectual work"*, because it cannot replicate our *reasoning* yet.

- Page 6 lines 96-97: "AI is a substitute for the innate ability of an individual player". I would soften a bit such a strong assertion. For example, "AI training reduces the importance of the innate ability of an individual player".
- Page 10 lines 177-178: "No female Shogi players can win through in Shoreikai and be promoted to become professional players". It is not clear from the exposition if women are not able to win because of their lack of skill, or if they are simply not allowed to participate in Shoreikai.
- Page 12 lines 203-206: I do not follow the reasoning why "the degree of dan is unlikely to reflect the players' strength". As I understand, this degree increases/decreases with good/bad performance. If *performance* is a proxy for *strength*, then why the degree of dan is not also a proxy for *strength*? All this is related with the phrase "Elo rating can be a player's ability that differs from performance (Bertoni et al. 2015)" found in lines 359-360, page 21. It is important that the authors clarify how performance and ability/strength differ.
- Page 15 line 257: "Its form is similar to the normal distribution for the periods …". The phrase in the text is not wrong, but it *suggests* normality. However, normality is not tested, and not directly needed, for the rest of the paper. I suggest avoiding mentioning normality. For example "We observe a unimodal distribution for the periods …". The same happens in page 34 lines 570-571.
- Page 16 line 279: "Sample covers ages over 45 and ...". I suggest changing it to "Sample covers players of all ages, and …".

Methodology:

- The Probit model should be better presented and interpreted, for instance …
- … in Page 21 lines 355-357: Instead of "$WIN_{igt} = \alpha_0 + \ldots + X'_{it}B + u_i$", it must say something like "$Prob[WIN_{igt} = 1] = F(\alpha_0 + \ldots + X'_{it}B)$, where F is the cumulative distribution function of the standard normal distribution".
- Page 22 lines 374-377: "Therefore, we use the winning … variable is 0 or 1.". This explanation should come before the functional form of the model presented in lines 355-357.
- My main problem with the use of Probit is that it is not clear the distinction between the coefficients of the model, and the marginal effects. In an OLS the two things are the same. But Probit is not linear (even without the quadratic terms in age) because of the cumulative function F. Specifically …
- … in Page 25 lines 419-420 it says that "Numbers without parentheses are marginal effects". I believe that this is not the case. I guess that in Table 2 you are showing the coefficients (alphas) of the equation in lines 355-357. What is important here is

to show which alphas are statistically significant, and their signs. But to interpret them as *marginal effects* is not correct. The marginal effect is the derivative of the Prob[WIN_{igt} = 1] with respect to one variable, fixing the rest at some specific values.

- Actually, it would be very desirable to report another table with the actual marginal effects, keeping the rest of variables at their mean values. These marginal effects then can be interpreted as the probability change for a given change in the respective variable.
- Page 25 lines 432: "winning rate is negative and linear". Nothing is linear in the Probit model (again, because of the function F). At most, you can say that "winning rate is decreasing regardless of the other variables". This would not be true if the coefficient of the quadratic age were statistically different from zero. Please, rephrase all your interpretations correctly.
- Page 27 line 457: change the name of the section from "Simulation" to something like "Predictions from the Probit model". This section is about the predicted probability of winning from the model estimated in the previous section. Therefore, avoid any mention to "simulation" through the whole paper.
- Page 30 line 517: "Panel A. Low innate ability". I think it should be 'High'.
- Page 31 line 518: "Panel B. High innate ability". I think it should be 'Low'.
- The explanations at lines 470 and 473 in page 28 are illustrative of the intuition behind the quadratic terms, but are not entirely accurate (because the function F is being ignored).
- Assuming that you are using Stata for your analysis, the following "toy" example illustrates what I am asking for:

```
probit WIN c.AGE##c.AGE c.DEB_AGE##c.DEB_AGE c.ELO c. ELO_OP   (and other vars.)

margins, dydx(*) atmeans

margins, dydx(DEB_AGE) at(DEB_AGE=(16(1)26))

marginsplot

margins, dydx(AGE) at(AGE=(20(1)60))

marginsplot
```

(please remember to put a i. in front of dummy/categorical variables, and c. in front of any continuous variable)

- Also, I would like to recommend you to explore the interaction between AGE and DEB_AGE (to see is the decline is different for different levels of innate ability). Something like this:

```
probit WIN c.AGE##c.AGE c.DEB_AGE##c.DEB_AGE c.AGE##c.DEB_AGE        (etc.)

margins, dydx(AGE) at(DEB_AGE=(16(1)26))

marginsplot
```

Exposition:

My first language is not English, but I think that most of the following suggestions will improve the readability of your paper. In addition, I strongly recommend that your next version be revised by an English native speaker.

- I would change "ageing declination" by "ageing decline" everywhere in the paper.
- In many cites there should be a space before the bracket. For example, in page 4 line 64 it should be "decreased [11]" instead of "decreased[11]".
- Page 3 line 53: change "professional Shogi player, Shin-ichi Sato" by "<u>the</u> professional Shogi player Shin-ichi Sato".
- Page 4 line 69: change "brain work" by "intellectual work".
- Page 5 line 80: change "others and use" by "others<u>, or</u> use".
- Page 5 line 89: "(Bertoni et al. 2015)" should be changed by "[17]" here, and all through the rest of the paper.
- Page 6 line 93: change "We construct game-level data from" by "We constructed a database of games from".
- Page 7 line 124: change "The difference in the rule is why …" by "Differences in the rules are the reason why …".
- Page 9 line 145: change "The higher grade is" by "The higher <u>the</u> grade is".
- Page 9 line 158: change "players could not survive and dropped out from" by "players cannot survive and drop out from".
- Page 11 line 185: change "Shogi players ordinary enter" by "Shogi players <u>usually</u> enter".
- Page 11 line 190: change "It at least takes 5 years to become" by "It takes at least 5 years to become".
- Page 12 line 199: change "of 'Ryuo'.Besides" by "of 'Ryuo'. Besides" (add the missing space).
- Page 15 lines 254-255: change the three cites to the "[#]" format (and elsewhere in the paper).
- Page 17 lines 293-294: change "debut years" by "debut age".
- Page 18 lines 309-311: change "In the Appendix, Figure A1 is illustrated by calculating the index. In Figure A1, we observed a similar …" by "In Figure A1 in the Appendix we can observe a similar …". (By the way, why and Appendix for only one figure and nothing else?)
- Page 20 lines 338, line 341, and also line 347: change "we proposed" by ""“we propose".
- Page 22 line 372: change "As seen in the function," by "As seen in the functional form of our model,".
- Page 22 line 380: change "increases the probability" by "increases the wining probability".
- Page 25 line 434: change "is rescued by" by "is <u>reduced</u> by".

- Page 28 lines 470 and also line 473: change A by ‘alpha’ and put the numbers as sub-indexes.
- Why Table 4 is on page 31 instead of page 27, before the Simulation section?
- Page 32 line 524: change “which are illustrated on the results” by “which illustrate the results”.
- Page 35 line 589: change “Shogi game becomes like” by “Shogi game has become like”.
- Page 36 line 601: change “other brain works” by “other intellectual tasks”.
