# Supplementary material for: AI, ageing and brain-work productivity: Technological change in professional Japanese chess
Source: PLoS One. 2024 May 2;19(5):e0299889. doi: 10.1371/journal.pone.0299889 (PMC11065245; doi:10.1371/journal.pone.0299889)
Supplement: Attachment — Submitted filename: PONE-D-23-24861_review2.pdf [file pone.0299889.s004.pdf]

The authors have satisfactorily addressed most of my previous observations. The writing is more natural and, as a consequence, the paper is now considerably more pleasant to read.

However, one important methodological aspect remains to be fixed, and it has to do with the marginal effects in the Probit model. To illustrate my point, let us start with the simplest OLS regression, with just one linear variable:

$$Y = \alpha + \beta_1 X + \varepsilon$$
$$E[Y \mid X] = \alpha + \beta_1 X$$
$$\frac{\partial E[Y \mid X]}{\partial X} = \beta_1 \quad \text{(marginal effect)}$$

Here the marginal effect is exactly equal to the corresponding β coefficient, regardless of the value of the explanatory variable *X*. But if we add a quadratic term:

$$Y = \alpha + \beta_1 X + \beta_2 X^2 + \varepsilon$$
$$E[Y \mid X] = \alpha + \beta_1 X + \beta_2 X^2$$
$$\frac{\partial E[Y \mid X]}{\partial X} = \beta_1 + 2\beta_2 X \quad \text{(marginal effect at } X\text{)}$$

then the marginal effect depends not only on two β coefficients, but also on the value of *X*. So, we must speak of "the marginal effect at the value *X*=*something*". Stata by default uses the average of all the observed values of *X* ('atmeans' in Stata language), but any other value can be specified with the 'margins' command.

In the case of the Probit model the problem is conceptually the same:

$$\Pr[Y = 1 \mid X] = F\left(\alpha + \beta_1 X + \beta_2 X^2\right)$$
$$\frac{\partial \Pr[Y = 1 \mid X]}{\partial X} = [\beta_1 + 2\beta_2 X] \times F'\left(\alpha + \beta_1 X + \beta_2 X^2\right) \quad \text{(marginal effect at } X\text{)}$$

Furthermore, the nonlinearity of the function *F* causes the same problem, even without the quadratic term:

$$\Pr[Y = 1 \mid X] = F\left(\alpha + \beta_1 X\right)$$
$$\frac{\partial \Pr[Y = 1 \mid X]}{\partial X} = \beta_1 \times F'\left(\alpha + \beta_1 X\right) \quad \text{(marginal effect at } X\text{)}$$

Thus, even in a "linear" Probit, we must speak of "marginal effect at *X*=*something*".

My problem is that in Table 2 of the paper the marginal effects of AGE and $AGE^2/100$ are both reported. This implies that you can change AGE while keeping $AGE^2/100$ constant (and vice versa), which is absurd. That is why I thought that, in the previous version, the authors were reporting the coefficients β_1, β_2, instead of the marginal effects.

I strongly recommend using the '##' operator in Stata, which is designed to treat quadratic terms in the proper way. For example:

```
probit WIN c.AGE##c.AGE
```

will be (internally) expand to:

```
probit WIN AGE AGE^2
```

The whole point here is that the 'margins' command will compute the derivatives knowing that AGE and AGE^2 cannot change independently. Therefore, the following commands will do the right thing:

```
margins, dydx(*) atmeans
margins, dydx(AGE) at(AGE=35)
margins, dydx(AGE) at(AGE=(20(1)60))
marginsplot
```

It is important to put an i. in front of dummy/categorical variables, and a c. in front of any continuous variable, so that the "derivatives" are computed in the right way by Stata.

Also, the authors should read these posts:

https://www.statalist.org/forums/forum/general-stata-discussion/general/1516972-probit-vs-dprobit

I am also worried about how Figures 7-12 have been computed. I assume that variables other than AGE have been replaced by their sample means in the corresponding subsample. But I am not sure if the normal cumulative function F was applied at the end. Stata can also help with that. For example:

```
probit WIN c.DEB_AGE##c.DEB_AGE
margins, at(DEB_AGE=(16(1)26))
marginsplot
```

should produce a graph similar to Figure 7.

To summarize, the authors must:

- Eliminate all squared variables from Table 1 (like $AGE^2/100$), because they are not independent variables.
- Correct Tables 2-4. In each case, I would show two tables instead of one: the actual β coefficients (the output of the 'probit' command), and then the marginal effects

(the output of the corresponding 'margins, dydx(*)' command). The first table contains the quadratic terms, but the second table does not.

- When reporting the marginal effects, it is required to also report at which values of the variables they have been computed (by default, at their means).
- Check that Figures 7-12 are generated correctly, taking into account all the nonlinearities of the Porbit model. As mentioned before, Stata 'margins' and 'marginsplot' commands can do that, but I do not know a simple way to overlap the predictions for the three time periods into a single graph. At least check that your figures are the same as the ones Stata produces.
- In page 24 lines 409-410 I would remove "The ordinary least squares (OLS), coefficients, and marginal effects are the same. However, the probit model used in this study differs.". As explained before, this is not true for all OLS regressions.
- In page 21 line 360: change "standard norm distribution" by "standard normal distribution".
- Revise all the explanations and interpretations resulting from the previous changes.
