# Supplementary material for: AI, ageing and brain-work productivity: Technological change in professional Japanese chess
Source: PLoS One. 2024 May 2;19(5):e0299889. doi: 10.1371/journal.pone.0299889 (PMC11065245; doi:10.1371/journal.pone.0299889)
Supplement: Attachment — Submitted filename: Reply_240201_notrack.docx [file pone.0299889.s005.docx]

PONE-D-23-24861R1

AI, Ageing and Brain-Work Productivity: Technological Change in Professional Japanese Chess

PLOS ONE

Dear Prof. Claudia Noemi González Brambila:

Thank you for your and the referees’ positive evaluations of this paper. We have revised and substantially improved the manuscript in accordance with the referees’ insightful comments and suggestions.

For your convenience, our replies to the referee are shown in blue.

The comments we received from you and Referee 2 were very helpful. We hope the reviewers find our replies to these comments satisfactory.

Best regards,

Eiji YAMAMURA

Reviewer #2:

One important methodological aspect remains to be fixed, and it has to do with the marginal effects in the Probit model.

Thank you for the detailed instructions on how to calculate the marginal effects of the probit model. I learned a lot and improved the quality of this paper through your comments. I hope you find the revision satisfactory. Below are our replies to your comments. For your convenience, the revised parts are colored red in the paper, and the replies to the referee are shown in blue. I have submitted the data and program files together with the revised manuscript and the figures files.

For your convenience, I have also numbered each comment.

1. Eliminate all squared variables from Table 1 (like AGE2/100), because they are not

independent variables.

Reply to 1: In compliance with this comment, we have deleted all the squared variables from Table 1.

2. Correct Tables 2-4. In each case, I would show two tables instead of one: the actual coefficients (the output of the ‘probit’ command), and then the marginal effects

(the output of the corresponding ‘margins, dydx(*)’ command). The first table

contains the quadratic terms, but the second table does not.

Reply to 2: The previous version of Table 4 consisted of Panels A and B. In the revised version, the contents of Table 4 have been divided into Tables 4 (a subsample of high ability) and 5 (a subsample of low ability). To meet the reviewer’s requirements, the new Tables 2-5 consist of Panels A (coefficients) and B (marginal effects).

3. When reporting the marginal effects, it is required to also report at which values of

the variables they have been computed (by default, at their means).

Reply to 3: The marginal effects of the variables on their means are reported, as suggested by the reviewer. In addition, we reported a marginal effect of “Age” at 35 years old. We added this sentence in the notes section below Tables 2-5, “In Panel B, the marginal effects are evaluated at mean values for the independent variables, except for the bottom line, where the marginal effect of Age is evaluated at 35 years old.”

.

4. Check that Figures 7-12 are generated correctly, taking into account all the

nonlinearities of the Porbit model. As mentioned before, Stata ‘margins’ and

‘marginsplot’ commands can do that, but I do not know a simple way to overlap the

predictions for the three time periods into a single graph. At least check that your

figures are the same as the ones Stata produces.

Reply to 4: Based on the newly estimated marginal effects on winning at various variable values, we calculated the “level of wins” to illustrate the new Figures 7-12. The program and dataset for the figures are attached.

5. In page 24 lines 409-410 I would remove “The ordinary least squares (OLS),

coefficients, and marginal effects are the same. However, the probit model used in

this study differs.”. As explained before, this is not true for all OLS regressions.

Reply to 5: Based on your comment, we have deleted this sentence.

6. In page 21 line 360: change “standard norm distribution” by “standard normal

distribution”.

Reply to 6: Thank you for pointing out this error. We fixed it.

7. Revise all the explanations and interpretations resulting from the previous changes

Reply to 7: We have revised the main body of the text to explain and interpret the new Tables 2-5, Figures 7-12, and Appendix Figures A2-A7. The main revisions are as follows:

(1) We explained how to calculate the marginal effect, which is heavily dependent upon your instructions (Lines 401-417; Lines 428-437).
(2) In 5.1., we interpreted the results of the Tables (Lines 493-503).

(3) A new interpretation of Figures 7-12 can be found in Section 5.2. (Lines 526-595).
